# Key factors of clinical research network capacity building

**DOI:** 10.1186/s40409-018-0152-0

**Published:** 2018-05-30

**Authors:** Guowei Li, Qianyu Wu, Yanling Jin, Thuva Vanniyasingam, Lehana Thabane

**Affiliations:** 1 Department of Health Research Methods, Evidence, and Impact, McMaster University, Hamilton, ON Canada; 2 Father Sean O’Sullivan Research Centre, St. Joseph’s Healthcare Hamilton, McMaster University, 3rd Floor Martha, Room H325, 50 Charlton Avenue E, Hamilton, ON L8N 4A6 Canada; 3 Centre for Evaluation of Medicines, Programs for Assessment of Technology in Health (PATH) Research Institute, McMaster University, Hamilton, ON Canada; 4 Department of Clinical Medicine, the First Clinical Medical College, Southern Medical University, Guangzhou, Guangdong Province China

**Keywords:** Capacity building, Clinical research, Network, Collaboration

## Abstract

In general, clinical research network capacity building refers to programs aimed at enhancing networks of researchers to conduct clinical research. Although in the literature there is a large body of research on how to develop and build capacity in clinical research networks, the conceptualizations and implementations remain controversial and challenging. Moreover, the experiences learnt from the past accomplishments and failures can assist in the future capacity building efforts to be more practical, effective and efficient. In this paper, we aim to provide an overview of capacity building in clinical research network by (1) identifying the key barriers to clinical research network capacity building, (2) providing insights into how to overcome those obstacles, and (3) sharing our experiences in collaborating with national and international partners to build capacity in clinical research networks. In conclusion, we have provided some insight into how to address the key factors of clinical research network capacity building and shared some empirical experiences. A successful capacity building practice requires a joint endeavor to procure sufficient resources and support from the relevant stakeholders, to ensure its efficiency, cost-effectiveness, and sustainability.

## Background

**Fig. 1:**
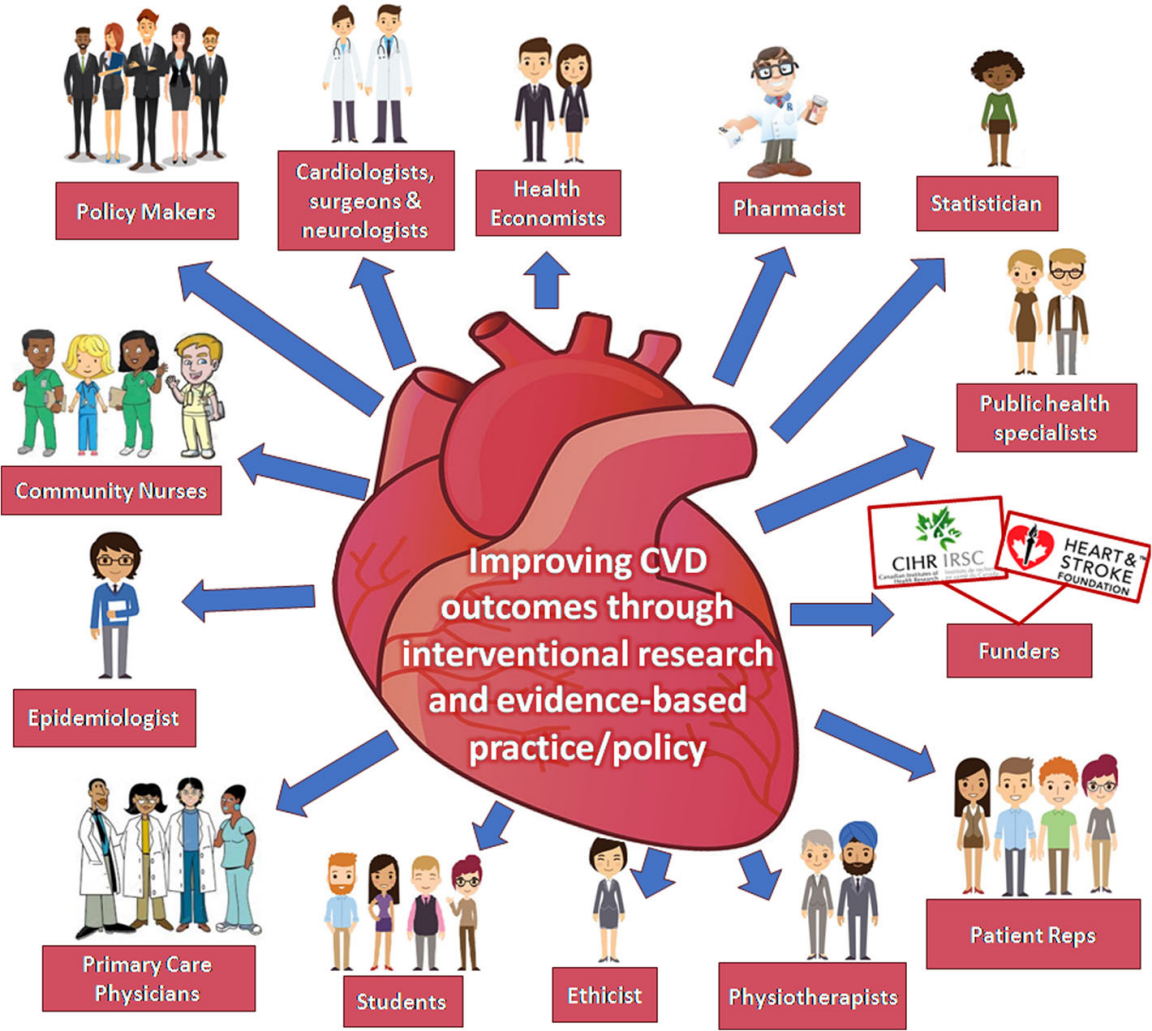
Example of a cardiovascular health research network

**Fig. 2:**
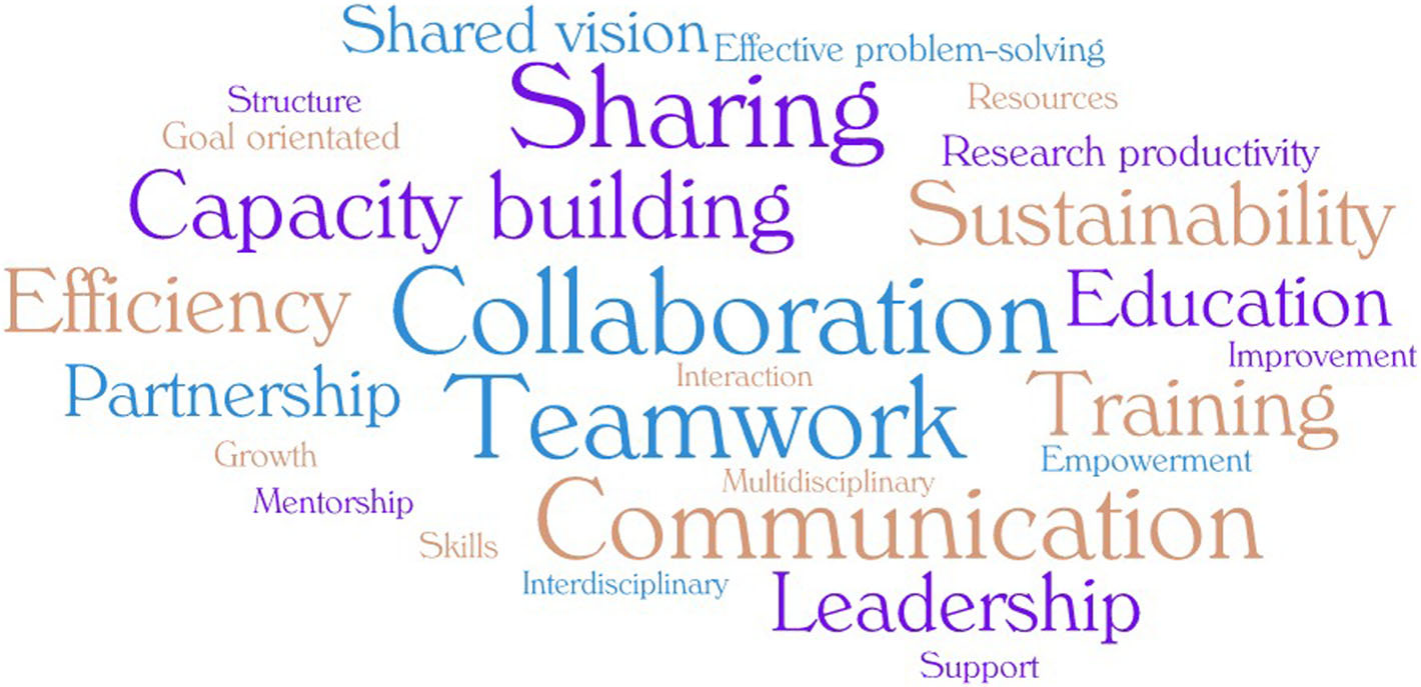
Word clouds of some key attributes of clinical research networks

The clinical research network is a joint and structured network of individuals, or institutions (such as universities, hospitals, institutes and other-related centers) that aims to (1) advance research and discovery of clinical studies, and (2) facilitate collaborations, educations and training, study implementations, data sharing and other research processes. Figure 1 shows an example of a research network composed of funders, policy makers, and individual researchers from different disciplines aimed at improving cardiovascular outcomes through interventional research, evidenced-based practice and policy. Some key attributes of research networks are presented in the word clouds (Fig. 2). These include collaboration, teamwork, communication, sharing, capacity building, sustainability, growth, mentorship, improvement, empowerment, education, support, and training, among others.

While capacity refers to the ability or power to finish, change, tackle, develop, or experience some objective or activity, capacity building indicates the interventions, procedures or activities aiming to produce sustained change or improvement to perform activities at levels of individuals, organizations, systems, national and/or international entities [1, 2]. Thus, clinical research network capacity building refers to programs aimed at enhancing networks of researchers to conduct clinical research. In the literature, there have been many examples of local, national and global clinical research networks with successful capacity development that perform and utilize health research resources efficiently, cost-effectively, and sustainably. Nevertheless, although there is a large body of research on how to develop and build capacity in clinical research networks, the conceptualizations and implementations remain controversial and challenging [2, 3]. The experiences learnt from the past accomplishments and failures can assist in the future capacity building efforts to be more practical, effective and efficient. Therefore, the objective of this paper is to provide an overview of capacity building in clinical research network by (1) identifying the key barriers to clinical research network capacity building, (2) providing insights into how to overcome those obstacles, and (3) sharing our experiences in collaborating with national and international partners to build capacity in clinical research networks.

### Key barriers to clinical research network capacity building

A recent systematic review has identified the key barriers to health research capacity development in low- and middle-income countries, which include [4]:

- fragmented research systems,
- insufficient funding,
- insufficient use of evidence,
- limited governance and regulatory capacity,
- insufficient networking,
- inefficient administration and management,
- inadequate material capacity,
- limited human capacity with knowledge and skills,
- limited practical experiences,
- lack of research leaders,
- lack of mentors and role models,
- lack of research culture,
- low motivation.

An extended literature search reveals similar barriers in developed countries [5]. In brief, the general critical hindrance to clinical research network capacity building is the lack of resources and support that include research system and networking, material support, regulation and management, evaluation, human resources, and evidence and experiences. A successful capacity building process will involve the joint efforts from all stakeholders to ensure sufficient and optimal resources and support.

### Some insights on how to overcome the key barriers to clinical research network capacity building

To address the key barriers, we propose five principles for good practice in clinical research network capacity strengthening. Table 1 shows the principles, corresponding examples of activities, barriers being addressed and some key resources for further reading. *The first principle is to understand the local context and accurately assess the existing research capacity*. This is a critical step as an initial evaluation of the current background and available baseline resources for the future realistic and feasible capacity building [6]. The first endeavor for successful capacity building processes generally requires comprehensive assessment of local situations or needs, strong partnership with local stakeholders to determine agenda, and identification of local champions or leaders.

**Table 1:** Insights on how to overcome the key barriers in building research network capacity

Principle	Examples of activities	Barriers being addressed	Key reference
Understand the local context and accurately assess the existing research capacity	Conduct an initial evaluation of the current background; Assess for available baseline resources; Evaluate feasibility of capacity building activities	Fragmented research systems; Insufficient use of evidence; Inefficient administration and management	[5, 6]
Use research evidence in practice and policy	Work towards integrating evidence into regulatory, legislative, and policy frameworks; Establish platforms to support evidence uptake at all levels; Foster dialogue and engagement between researchers and users of research evidence	Insufficient use of evidence; Limited practical experiences; Lack of research culture; Limited governance and regulatory capacity	[4, 5]
Secure sufficient collaboration and network and establish strong partnership with funders	Build local, national and international collaborative network by involving numerous hospitals and clinical centers; Develop collaborative communication; Advance experience-sharing processes; Procure long-term and joint funding	Fragmented research systems; Insufficient networking; Insufficient funding; Limited practical experiences	[7, 8]
Increase human resources factors including the supervision and mentorship, and the skills and experiences	Foster and incentivize collaborations; Establish platforms for exchange of ideas and cross-fertilization; Create strong supervision and mentorship systems; Expand collaborative network to secure maximum human resources support; Enhance experience- and skill-sharing activities	Fragmented research systems; Insufficient networking; Limited practical experiences; Limited human capacity with knowledge and skills; Lack of mentors and role models	[9, 10]
Identify institutional leadership and environment evaluation	Support research infrastructure; Align incentives or rewards with institutional goals and curricula with local and (inter)national needs; Foster co-op or experiential learning	Fragmented research systems; Lack of research leaders; Low motivation; Inadequate material capacity	[11, 12]

*A second principle is to use the most up-to-date research evidence in practice and policy*. Building capacity in use of evidence among practitioners and policy-makers is a critical component of successful capacity building [4]. In general, use of research evidence needs to work towards integrating evidence into regulatory, legislative, and policy frameworks, establish platforms to support evidence uptake at all levels, and foster dialogue and engagement between researchers and users of research evidence.

*Thirdly, one would secure sufficient collaboration and networking for effective communication and experience sharing and to establish strong partnership with funders.* For example, the CANadian Network and Centre for Trials Internationally (CANNeCTIN) is a national network for Canadian-led trials in cardiovascular diseases and diabetes mellitus and is funded by Canadian Institute of Health Research (CIHR) [7]. The CANNeCTIN involves a collaborative Canadian network and an expanded international network of numerous hospitals and clinical centers. The stable funding and networking support forms the sound base to conduct nation- and internationally-wide trials via the long-term collaborative communication and experience-sharing processes. A capacity building endeavor pertains to the flexible approach and sustaining progress at multiple levels, among which funding is a major issue in many capacity building cases [8]. How to procure long-term and joint funding for continued capacity building needs a strategic and thoughtful plan that will lead to sustainability. Engaging funding partners in understanding benefits of research to society, working with funders to determine funding priorities, building capacity to support peer-review process for funding initiatives, and working with media to mobilize public support for research may be worthwhile practices to ensure stable funding in capacity building processes.

*A fourth principle is related to the human resource factors including the supervision and mentorship, and the skills and experiences.* Such factors have been identified as a significant role across a range of research network capacity building endeavors [9]. A successful capacity building process will require (1) enhancing research productivity, fostering and incentivizing collaborations, and establishing platforms for exchange of ideas and cross-fertilization; (2) strong supervision and mentorship that can facilitate securing more resources, train and educate students or junior researchers for sustaining improvement, and monitor and evaluate the capacity building efforts; and (3) sufficient skills and experiences that can ensure the effectiveness and efficiency of capacity development [4, 6, 10].

*A last principle involves identifying institutional leadership and environment evaluation.* This step may help with support research infrastructure, align incentives or rewards with institutional goals and curricula with local and (inter) national needs, and foster co-op or experiential learning. For instance, lack of research infrastructure is a common barrier to capacity building [11]. Exemplary good practices include (1) enabling environments for joint appointments between disciplines and research centers with the purpose of developing new curricula, task groups, research teams, studentships and fellowships; (2) ensuring credits and promotions of working from clinical research networks, and (3) building an interactive context with the combination of operational base, synergistic structure and cumulative structure [11, 12]. The INDOX (INDia-Oxford) Cancer Research Network is an exemplary case of research network capacity development [13]. It uses the base of India’s top nine cancer centers as the leadership to proactively collaborate with University of Oxford for research conductions, fosters experiential learning, and promotes an interactive platform for communications and sharing.

### Some empirical Canadian-based experiences of clinical research network capacity building

#### The African Development AIDS Prevention Trials capacity (ADAPT) program

Led by the Centro de Investigación de Enfermedades Tropicales (CIET) and funded by the International Development Research Centre through their HIV Prevention Trials Capacity Building Grants program of the Global Health Research Initiative in Canada, the ADAPT program involved capacity-building in HIV trials in ten sub-Saharan countries that included Botswana, Lesotho, Malawi, Mozambique, Namibia, Swaziland, Zambia and Zimbabwe, South Africa, and Tanzania [14]. The global goal of the ADAPT program was to develop state-of-the-art, autonomous, and sustainable health measurement and planning resources for African countries to better implement and evaluate HIV and AIDS prevention interventions.

The specific aims of the program included: (1) to increase the capacity of the individual African researchers; (2) to increase the capacity of the African institutions; (3) to establish a framework for an African-led, multi-country AIDS prevention trial; and (4) to facilitate the development of a multi-country AIDS prevention trial. The first phase of ADAPT took place in 2007–2009 and included an 8 week course for African researchers focused on randomized controlled trials (RCTs) and the use of epidemiology for planning. This provided the foundation for the network of researchers created across the region who continued to work with CIET, to design and implement an AIDS prevention trial through interventions to reduce choice-disablement and gender-based violence in three of the countries (Botswana, Namibia, and Swaziland). The second phase of the ADAPT program took place in 2010–2014. During this phase, the program sponsored two people to enroll into Masters programs at the University of the Witwatersrand and the University of Pretoria in South Africa, and four others to undertake Bachelors programs (at the University of Namibia and University of South Africa).

In Botswana, almost all the members of parliament who were contacted considered the training for making healthcare policies as crucial. In October 2011, the ADAPT program collaborated with the government of Botswana to run a 2-day training session for parliamentarians about evidence-based decision making with a further training session took place over 2 days in November 2012. The training program for parliamentarians covered a broad range of evidence-based training including how to appraise control group, the influence of bias, significance, the number needed to treat, and cost-effectiveness, among others. The program secured sufficient training support successfully including networking, funding, local and international supervision, and other related resources. After the training, these members of parliament had a better understanding of how to allocate budgets for the specific projects, what population would benefit from the projects, and what existing evidence could be used and what evidence gaps should be addressed. Instead of sitting back and just granting funding, well-educated decision-makers could be proactively involved in the developing phases of the research. The success of the ADAPT program in Botswana had interested the decision-makers and other relevant researchers for further sustained training sessions [15].

#### The Canadian trials network (CTN) HIV workshop

The CTN for HIV research, funded by CIHR, is committed to developing treatments, vaccines and a cure for HIV disease and AIDS by conducting scientifically sound and ethical clinical trials [16]. The HIV workshop provided by the CTN for HIV research is another exemplary case of clinical research network capacity building. The CTN, together with the International Conference on AIDS and Sexually Transmitted Infections in Africa (ICASA), organized the workshop to share the CTN’s missions and experiences with the junior African HIV researchers, address the ethical challenges in conducting the HIV studies, and help enhance the career development and project collaborations for the young researchers. The workshop attracted substantially more researchers than expected; and the capacity building in ethical and educational strategies in HIV-related research received highly positive feedback at the ICASA conference [17].

#### The Drug Safety and Effectiveness Cross-disciplinary Training (DSECT) program

The one-year DSECT program is supported and organized by the CIHR, McMaster University, St. Joseph’s Healthcare Hamilton, and other academic entities in Canada [18]. The program, funded by CIHR, aims to provide fundamental knowledge on drug safety and effectiveness, build scientific bridge across different domains (trainee and investigator), develop collaborative opportunity for trainees through practical projects, and incorporate knowledge translation in four different domains of sciences (biosciences, clinical therapeutics, population health and epidemiology, and health services and policy research). The curriculum of the DSECT program contains an annual symposium, a series of online synchronous lectures and tutorials, a one-to-one paired mentor, online self-study modules, online discussion sessions, practical sessions, an Objective Structured Knowledge Translation Experience (OSKTE), and a book club. With other trainees from other disciplines and the paired mentor, trainees can build their capacity for effective communication and collaboration skills. It is also expected that trainees should be able to enhance their knowledge and appraisal of drug safety and effectiveness information independently.

The DSECT program has been proven a highly appreciated training platform and has obtained long-term funding for its sustainability. Although some challenges exist including the unavailability of mentors due to their busy schedules, relatively high work load for trainees, the short length of the program, and unexpected technology shortcomings, the DSECT program has been significantly improved by procuring more resources and support and by developing more flexible individual education and learning plans [19].

#### The Africa Center for Biostatistical Excellence (ACBE) initiative

Well-educated biostatistical methodologists who are sophisticated in study design, implementation, data analyzing, and results reporting are significantly lacking in the sub-Saharan African region. The ACBE, funded by the National Institutes of Health (NIH), was therefore proposed to reduce such shortage and to enhance the biostatistics capacity. The ACBE had secured support and resources for its foundation, with a collaborative effort across academic and research institutions. The connections between Canadian and local academia ensured the human resource support for its sustainability and efficiency. The ACBE will act as a vehicle for promoting biostatistics capacity building through specialized academic Master of Science (MSc) programs and regular workshops targeting researchers. The ACBE aimed to sustainably produce qualified biostatistical researchers who can be able to independently tackle the research difficulties and challenges in Africa [20, 21].

### Common key elements learnt from the empirical Canadian-based experiences

The common key elements, learnt from the aforementioned examples, are summarized in Table 2. The key elements to ensure a successful capacity building process generally include: (1) program-level-based elements that are composed of leadership, local contextual assessment, mentorship, collaboration, and partnership with funders; and (2) individual-level-based components that consist of commitment, compassion, sharing, openness, patience, and friendship. These elements may significantly help facilitate the achievement of the clinical research network capacity building.

**Table 2:** Common key elements learnt from the empirical Canadian-based experiences

Program-level-based	Individual-level-based
Strong local leadership; Comprehensive local contextual assessment; Good mentorship; Sufficient collaboration; Strong partnership with funders	Passion (commitment); Compassion (empathy); Sharing (unselfishness); Openness (transparency); Humility (patience); Friendship (fun)

## Conclusions

In this paper, we have provided some insight into how to address the key factors of clinical research network capacity building and shared some empirical experiences. A successful capacity building practice requires a joint endeavor to procure sufficient resources and support from the relevant stake-holders, to ensure its efficiency, cost-effectiveness, and sustainability.

## Abbreviations

ACBE: Africa center for Biostatistical excellence
ADAPT: African Development AIDS Prevention Trials
CANNeCTIN: Canadian network and centre for trials internationally
CIET: Centro de Investigación de Enfermedades Tropicales
CIHR: Canadian Institute of Health Research
CTN: Canadian trials network
DSECT: Drug Safety and Effectiveness Cross-disciplinary Training
ICASA: International Conference on AIDS and Sexually Transmitted Infections in Africa
INDOX: INDia-Oxford
MSc: Master of Science
NIH: National Institutes of Health
OSKTE: Objective Structured Knowledge Translation Experience
RCT: Randomized controlled trial
